# Mind the Gap: New Full-Length Sequences of *Blastocystis* Subtypes Generated via Oxford Nanopore Minion Sequencing Allow for Comparisons between Full-Length and Partial Sequences of the Small Subunit of the Ribosomal RNA Gene

**DOI:** 10.3390/microorganisms9050997

**Published:** 2021-05-05

**Authors:** Jenny G. Maloney, Monica Santin

**Affiliations:** Environmental Microbial and Food Safety Laboratory, Agricultural Research Service, United States Department of Agriculture, Beltsville, MD 20705, USA; jenny.maloney@usda.gov

**Keywords:** *Blastocystis*, long-read sequencing, MinION, ribosomal RNA, subtypes

## Abstract

*Blastocystis* is a common food- and water-borne intestinal protist parasite of humans and many other animals. *Blastocystis* comprises multiple subtypes (STs) based on variability within the small subunit ribosomal (*SSU* rRNA) RNA gene. Though full-length reference sequences of the *SSU* rRNA gene are a current requirement to name a novel *Blastocystis* subtype, full-length reference sequences are not currently available for all subtypes. In the present study, Oxford Nanopore MinION long-read sequencing was employed to generate full-length *SSU* rRNA sequences for seven new *Blastocystis* subtypes for which no full-length references currently exist: ST21, ST23, ST24, ST25, ST26, ST27, and ST28. Phylogenetic analyses and pairwise distance matrixes were used to compare full-length and partial sequences of the two regions that are most commonly used for subtyping. Analyses included *Blastocystis* nucleotide sequences obtained in this study (ST21 and ST23–ST28) and existing subtypes for which full-length reference sequences were available (ST1–ST17 and ST29). The relationships and sequence variance between new and existing subtypes observed in analyses of different portions of the *SSU* rRNA gene are discussed. The full-length *SSU* rRNA reference sequences generated in this study provide essential new data to study and understand the relationships between the genetic complexity of *Blastocystis* and its host specificity, pathogenicity, and epidemiology.

## 1. Introduction

*Blastocystis* sp. is one of the most common intestinal parasites observed in humans and has a global distribution [1,2]. It is spread via the fecal–oral route, with contaminated food and water being likely means of transmission [3,4,5]. While human infection has been associated with both gastrointestinal and extraintestinal symptoms, asymptomatic carriage is quite common, thus making the relationship between infection and disease unclear [6,7,8]. *Blastocystis* is also commonly observed in animal hosts, and while studies on symptomatic infection in animals are lacking, there is some evidence to support the zoonotic transmission of *Blastocystis* between humans and animals [2].

One factor thought to be key in explaining the pathogenicity and zoonotic potential of *Blastocystis* is the existence of a remarkable degree of genetic variability within the genus. This genetic variability has thus far been largely described using variability within the small subunit ribosomal RNA gene (*SSU* rRNA gene). Based on differences within the *SSU* rRNA gene, *Blastocystis* is divided into genetic groupings called subtypes. Currently, subtype designations are assigned using a numbering scheme that is chronological and based on publication order [9].

To be considered a candidate for a novel subtype designation, it is suggested in current proposed guidelines that a sequence should meet several inclusion criteria including: (1) one must be able to obtain the near full-length *SSU* rRNA gene (≥80% of the approximately 1800 bp); (2) one must demonstrate that the novel subtype sequence differs from any existing subtype by at least 4%; (3) one must inspect novel subtype sequences for evidence of chimeras; and (4) one must perform phylogenetic analysis to ensure that new subtypes do not cluster with previously existing subtypes [9].

Of the 29 proposed subtypes, there are 25 that meet the current recommended criteria for unique subtype designations. The status of 22 of these subtypes (ST1–ST17, ST21, and ST23–ST26) was recently reviewed, while the other three subtypes (ST27–ST29) were first identified and proposed as novel subtypes after the publication of the review [9,10,11]. Furthermore, several published subtypes have been proposed to be either rejected or tentatively accepted based on their failure to meet the proposed subtype-naming criteria. These include subtypes ST18, ST19, ST20, and ST22, which are suggested to be invalid because they have the appearance of being potentially chimeric [9]. While subtypes ST21, ST23, ST24, ST25, and ST26 were suggested to be tentatively accepted [9]. These subtypes were named using a fragment of the *SSU* rRNA gene, but no full-length sequence has been generated to confirm their validity [12]. There are also two recently proposed subtypes, ST27 and ST28, which do not currently have published full-length reference sequences [10].

Historically, a major hurdle for full-length *SSU* rRNA sequence generation has been a lack of methodology that can be used to generate full-length sequences from the often-complex sample matrixes from which DNA for *Blastocystis* is isolated. Thus, standard primers that can amplify a small region of the gene are used for screening samples, and positive samples are later sequenced to identify subtypes [13,14]. These primers are both sensitive and specific, and they can identify all named subtypes and potentially novel subtypes. However, to achieve full-length sequences of the *SSU* rRNA gene, scientists within this field have had to use less specific primers that generate a longer sequence or attempt to piece the gene together using multiple primer sets that may contribute to the generation of chimeric and/or artifactual sequences [15]. A recent study demonstrated the suitability of Oxford Nanopore MinION long-read sequencing for the generation of accurate full-length sequences of *Blastocystis*, thus providing a new method for addressing some of the issues surrounding subtype designations [16].

**Figure 1:**
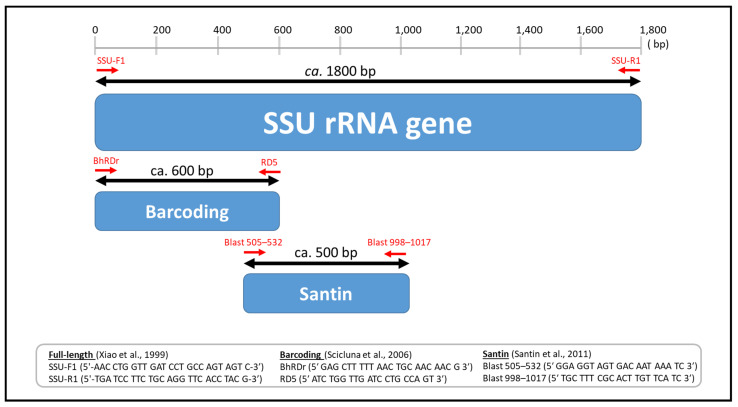
Diagram showing small subunit ribosomal RNA (*SSU* rRNA) gene indicating regions amplified by barcoding and Santin primers as well as information on primers used to amplify all gene segments used in this study.

As reference sequences are integral to the validity of the current subtype system, full-length sequences of all currently accepted *Blastocystis* subtypes are needed. In the present study, MinION sequencing was used to generate full-length reference sequences for *Blastocystis* subtypes ST21, ST23, ST24, ST25, ST26, ST27, and ST28—for which no published full-length sequences currently exist. Phylogenetic and pairwise distance analyses were performed by incorporating available full-length reference sequences of the *SSU* rRNA gene for all known *Blastocystis* subtypes, as well as sequences for ST21 and ST23–ST28 that were generated in this study. Additionally, analyses were conducted for sequence regions of the *SSU* rRNA gene amplified by the two most common standard primer sets used for amplifying and sequencing *Blastocystis* in survey studies [13,14] (Figure 1). This information provides reference sequences that are essential for understanding the current status of *Blastocystis* subtype relationships and that can be used as a starting point to propose or describe novel subtypes in future studies.

## 2. Materials and Methods

### 2.1. Source of Blastocystis Isolates

Five DNA samples containing *Blastocystis* obtained from animal fecal isolates were used in this study (Table 1). All isolates used in this study had been previously typed using Sanger and/or next generation sequencing using previously reported protocols [12,13]. We selected samples that had been identified as containing *Blastocystis* subtypes ST21 and ST23–ST28 for which no published full-length sequences are currently available.

### 2.2. PCR Amplification of the Full-Length SSU rRNA Gene

The approximately 1800 base pair *SSU* rRNA gene was amplified by PCR using a previously described Nanopore sequencing strategy [15]. Briefly, a PCR using SSU-F1 (5’-AAC CTG GTT GAT CCT GCC AGT AGT C-3’) and SSU-R1 (5’-TGA TCC TTC TGC AGG TTC ACC TAC G-3’) that amplifies the SSU rRNA gene of most eukaryotic organisms was performed [17]. Each reaction used 1 µM forward and reverse primers, as well as 12.5 µL of KAPA HiFi HotStart ReadyMix (KAPABioSystems, Cape Town, South Africa), in a 25 µL reaction volume. Initial denaturation was performed at 98 °C for 5 min, followed by 35 cycles of amplification (20 sec at 98 °C, 45 sec at 60 °C, and 90 sec at 72 °C) and final extension for 5 min at 72 °C. Following amplification, amplicons were visualized using a QIAxcel (Qiagen, Valencia, CA, USA) and quantified using a Qubit fluorometer (ThermoFisher Scientific, Waltham, MA, USA).

The Nanopore sequencing library was prepared using the Oxford Nanopore Technologies (ONT) SQK-LSK109 Ligation Sequencing Kit (ONT, Oxford, UK) following the manufacturer’s protocol for Amplicons by Ligation (ACDE_9064_v109_revQ_14Aug2019). Amplicons were quantified and diluted to ensure that 150 fmol of DNA were used as input into library prep, as recommended by the protocol. The nanopore library was run on an R9.4 flow cell (FLO-MIN106) using an ONT MinION Mk1B and MinKNOW v20.06.15 software. Basecalling was performed using ONT Guppy v4.0.11 (gpu) using a minimum quality score cut off of 7 for filtering low-quality reads. FASTQ reads were also length-filtered to only include reads between 1700 and 2000 nucleotides. Reads were then corrected using canu v2.1, and consensus sequences were generated by clustering reads using the vsearch—cluster_fast command (vsearch v2.14.1) with a 98% identity threshold, checked for chimeras, and polished as previously described [16].

For comparison purposes, for each same sample, full-length sequences and partial sequences obtained with MinION and MiSeq, respectively, were aligned using ClustalW in MegAlign 15 (DNASTAR Lasergene 15, Madison, WI, USA), and pairwise distances between consensus sequences were calculated. The nucleotide sequences generated in this study were deposited in GenBank under the accession numbers MW887928–MW887935.

### 2.3. Phylogenetic and Pairwise Distance Analyses

The full-length *SSU* rRNA gene nucleotide sequences obtained in this study, appropriate full-length *Blastocystis* reference nucleotide sequences obtained from the reference database found at http://entamoeba.lshtm.ac.uk/blastorefseqs.htm (accessed on 17 March 2021), and other full-length sequences available in GenBank were included to generate a phylogenetic tree rooted using *Proteromonas lacertae*, a Stramenopile (which is closely related to *Blastocystis*) as an outgroup (Table 2). Nucleotide sequences were aligned with the Clustal W algorithm using MEGA X, and phylogenetic analyses were performed using neighbor-joining (NJ) and maximum-likelihood (ML) methods and genetic distances calculated with the Kimura 2-parameter model using MEGA X [18,19]. Because the 64 nucleotide sequences used in this study differ in length, ends of sequences were trimmed. There were a total of 1953 positions in the final dataset. Bootstrapping with 1000 replicates was used to determine support for the generated clades. Evolutionary analysis was conducted to establish divergence between sequences (pairwise distance) using the Kimura 2-parameter model in MEGA X [18,19].

For the purpose of comparison, analyses employing the same 64 nucleotide sequences utilized for full-length analyses were conducted for those regions of the *SSU* rRNA gene amplified by the two most common standard primers sets used for amplifying and sequencing *Blastocystis* in survey studies [13,14] (Figure 1). There were a total of 587 and 568 positions in the final datasets for the barcoding and Santin regions, respectively.

## 3. Results

### 3.1. Full-Length SSU rRNA Gene for ST21, ST23, ST24, ST25, ST26, ST27, and ST28

The five samples included in this study were selected based on the results of screening via MiSeq sequencing of an approximately 500 bp region of the *SSU* rRNA gene and were found to contain ST21, ST23, ST24, ST25, ST26, ST27, and/or ST28. Nanopore sequencing was performed to produce full-length *SSU* rRNA gene sequences for these seven *Blastocystis* subtypes because no full-length references were available. Full length-sequences were successfully obtained for all seven subtypes for which no full-length reference sequences currently exist. Multiple subtypes were obtained from three of the five samples with ST21 and ST24, ST23 and ST26, and ST27 and ST28 being present in the same sample (Table 1). Agreement between the MiSeq sequence and the MinION sequence for each subtype was high, ranging from 99.6 to 100% for all sequences (Table 1).

### 3.2. Phylogenetic Analyses

**Figure 2:**
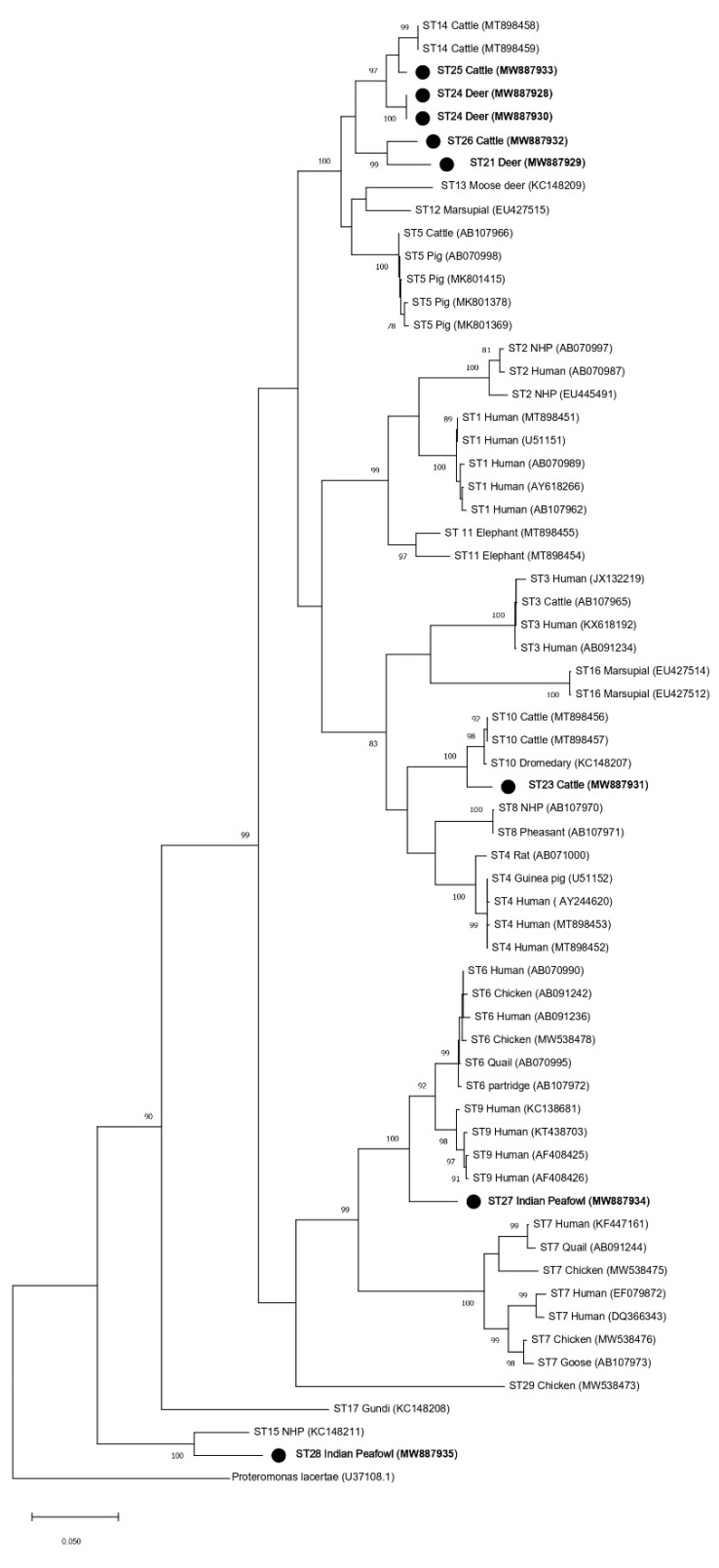
Phylogenetic relationships among *Blastocystis* full-length *SSU* rRNA gene nucleotide sequences generated in the present study (represented with a black filled circle) and representative reference sequences of the accepted subtypes (Table 2). *Proteromonas lacertae* was used as outgroup taxon to root the tree. Analysis was conducted by a maximum likelihood method. Genetic distances were calculated using the Kimura two-parameter model. This analysis involved 64 nucleotide sequences, and there were a total of 1953 positions in the final dataset. Bootstrap values lower than 75% are not displayed.

**Figure 3:**
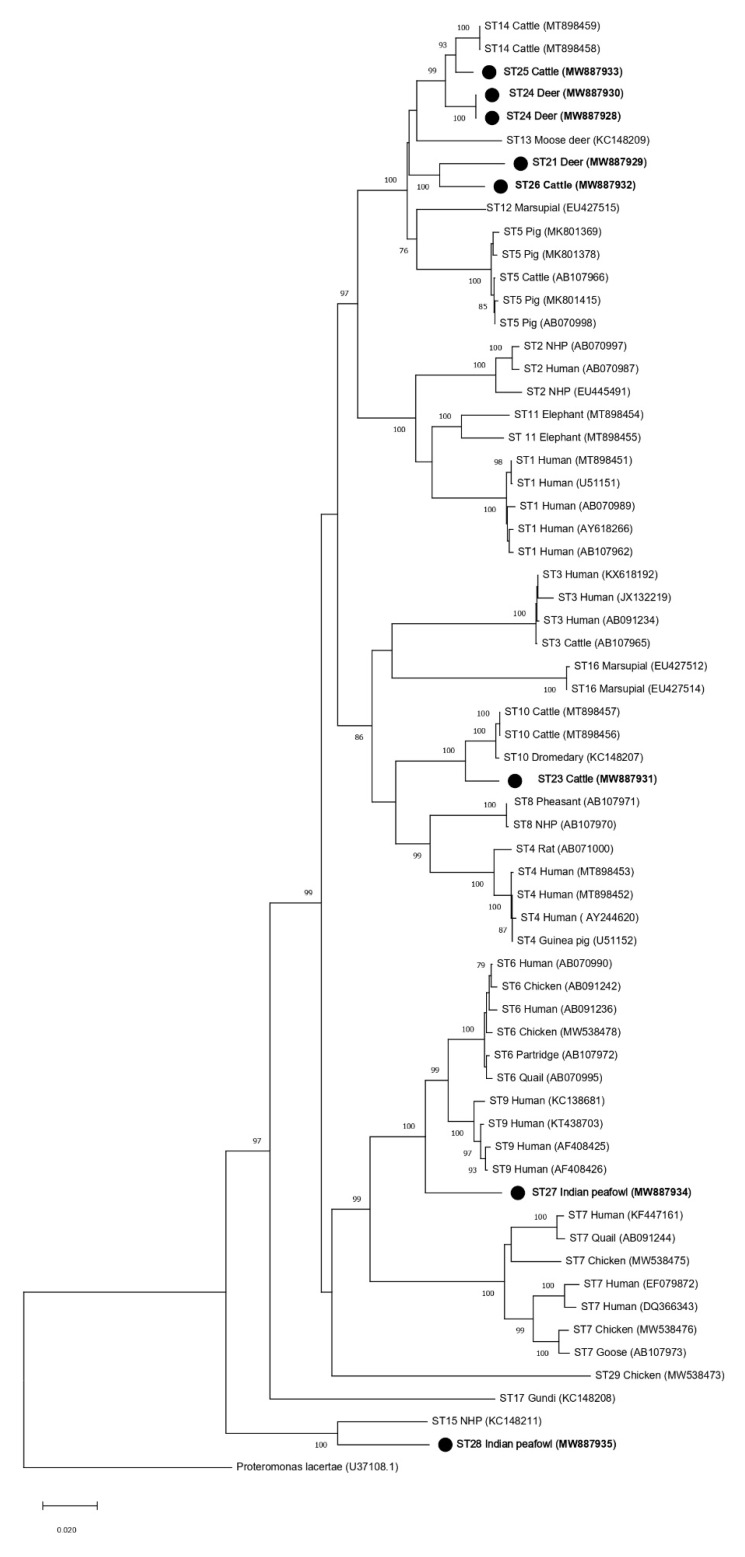
Phylogenetic relationships among *Blastocystis* full-length *SSU* rRNA gene nucleotide sequences generated in the present study (represented with a black filled circle) and representative reference sequences of the accepted subtypes (Table 2). *Proteromonas lacertae* was used as outgroup taxon to root the tree. Analysis was conducted by a neighbor-joining method. Genetic distances were calculated using the Kimura two-parameter model. This analysis involved 64 nucleotide sequences, and there were a total of 1953 positions in the final dataset. Bootstrap values lower than 75% are not displayed.

Phylogenetic analyses were performed to generate trees using full-length *SSU* rRNA gene sequences from this study and references sequences for all other accepted *Blastocystis* subtypes (ST1–ST17, ST21, and ST23–ST29) with *Proteromonas lacertae* included as an outgroup (Table 2). Both the ML and NJ phylogenetic trees generated using the full-length MinION sequences reported in this study (ST21, ST23–ST27) showed similar topologies (Figure 2 and Figure 3). The subtype clustering observed here was similar to prior ML analyses performed for subtypes ST1–ST10 and ST13–ST17 using full-length sequences (no full-length references were available for ST11 and ST12 at that time) [15]. However, in the present analysis, ST16 no longer formed an external branch and instead nested within a clade formed with ST3 (Figure 2 and Figure 3). Clades formed by ST23, ST27, and ST28 were supported by a bootstrap proportion of 100 by both NJ and ML. Clades formed by ST21 and ST26 were supported by a bootstrap proportion of 99 and 100 for ML and NJ, respectively. ST25 formed a clade with ST14 with bootstrap support of 97 and 99 for ML and NJ, respectively. The bootstrap support values for clades ST14 and ST25 were 45 and 93 for ML and NJ, respectively.

**Figure 4:**
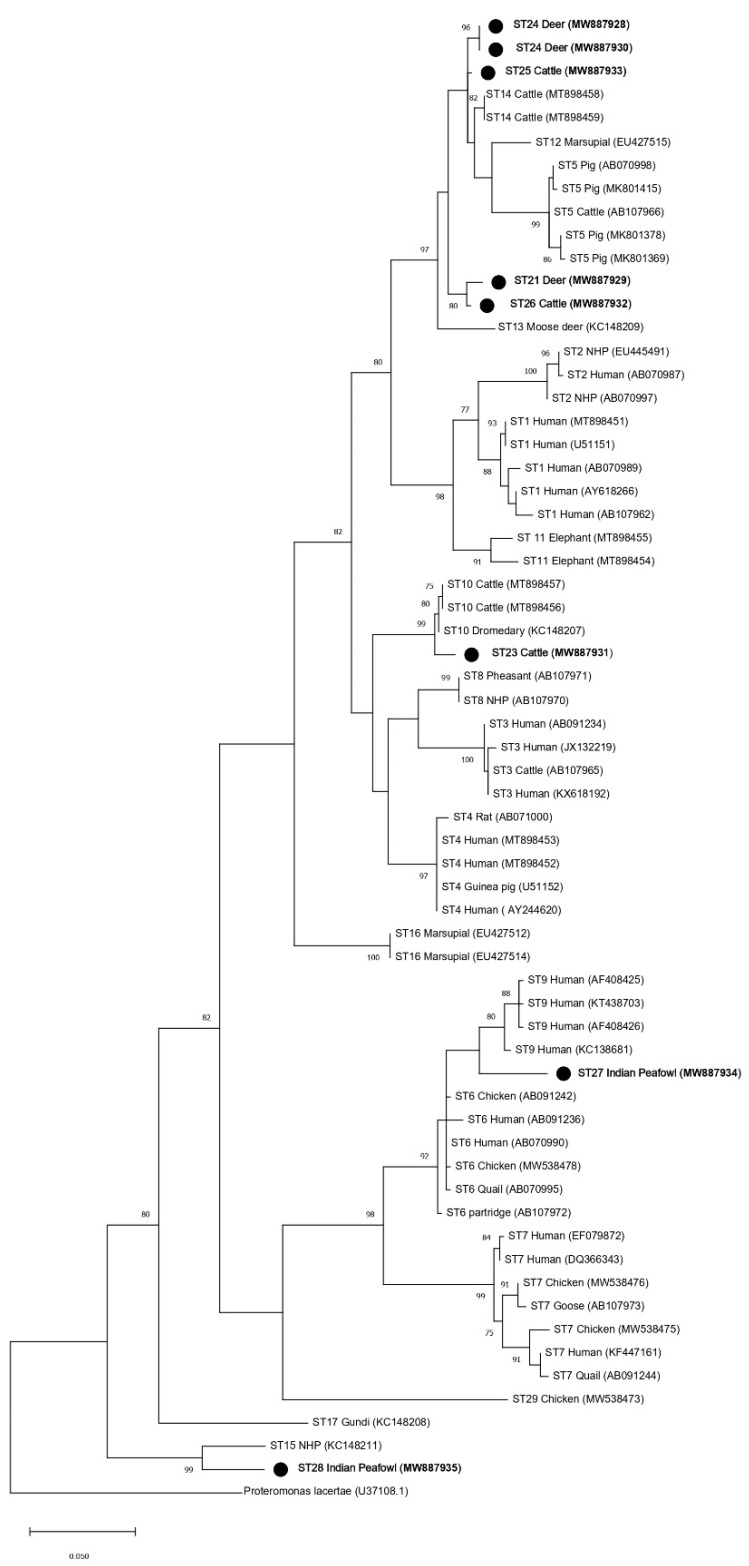
Phylogenetic relationships among *Blastocystis* partial *SSU* rRNA gene sequences of the barcoding region generated in the present study (represented with a black filled circle) and representative reference sequences of the accepted subtypes (Table 2). *Proteromonas lacertae* was used as outgroup taxon to root the tree. Analysis was conducted by a maximum likelihood method. Genetic distances were calculated using the Kimura two-parameter model. This analysis involved 64 nucleotide sequences, and there were a total of 587 positions in the final dataset. Bootstrap values lower than 75% are not displayed.

**Figure 5:**
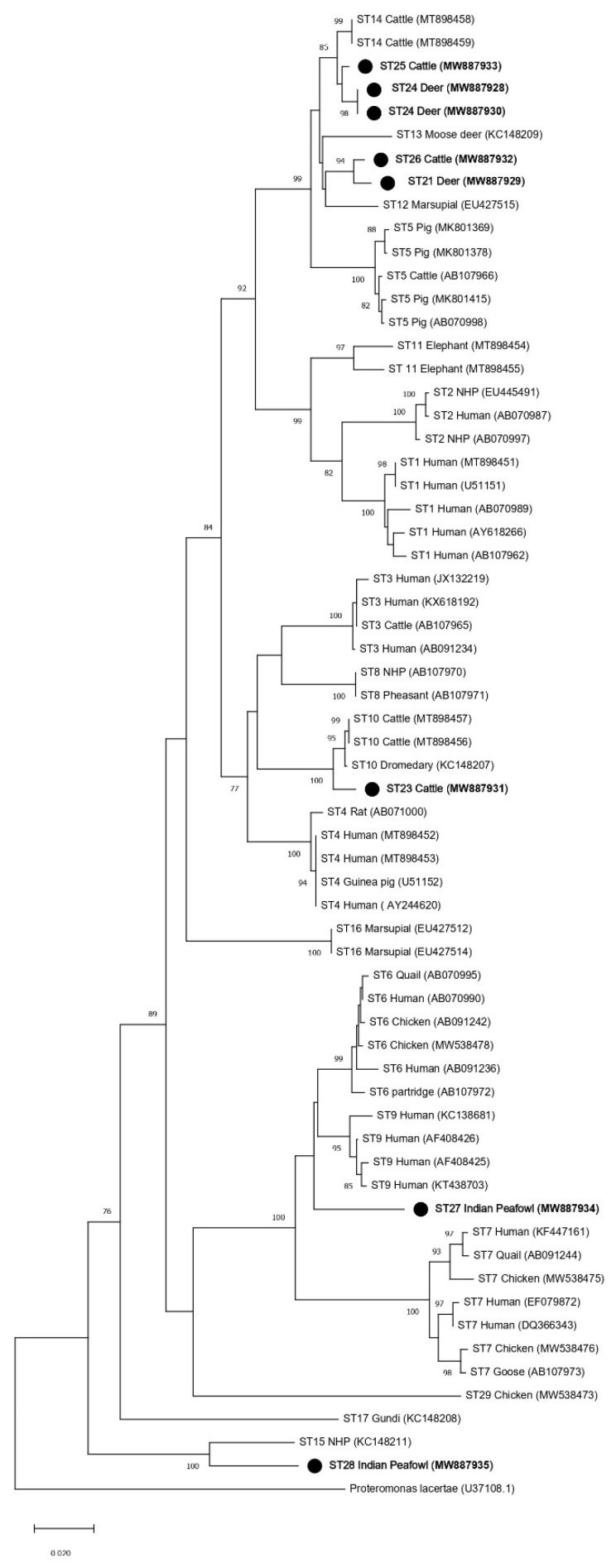
Phylogenetic relationships among *Blastocystis* partial *SSU* rRNA gene sequences of the barcoding region generated in the present study (represented with a black filled circle) and representative reference sequences of the accepted subtypes (Table 2). *Proteromonas lacertae* was used as outgroup taxon to root the tree. Analysis was conducted by a neighbor-joining method. Genetic distances were calculated using the Kimura two-parameter model. This analysis involved 64 nucleotide sequences, and there were a total of 587 positions in the final dataset. Bootstrap values lower than 75% are not displayed.

**Figure 6:**
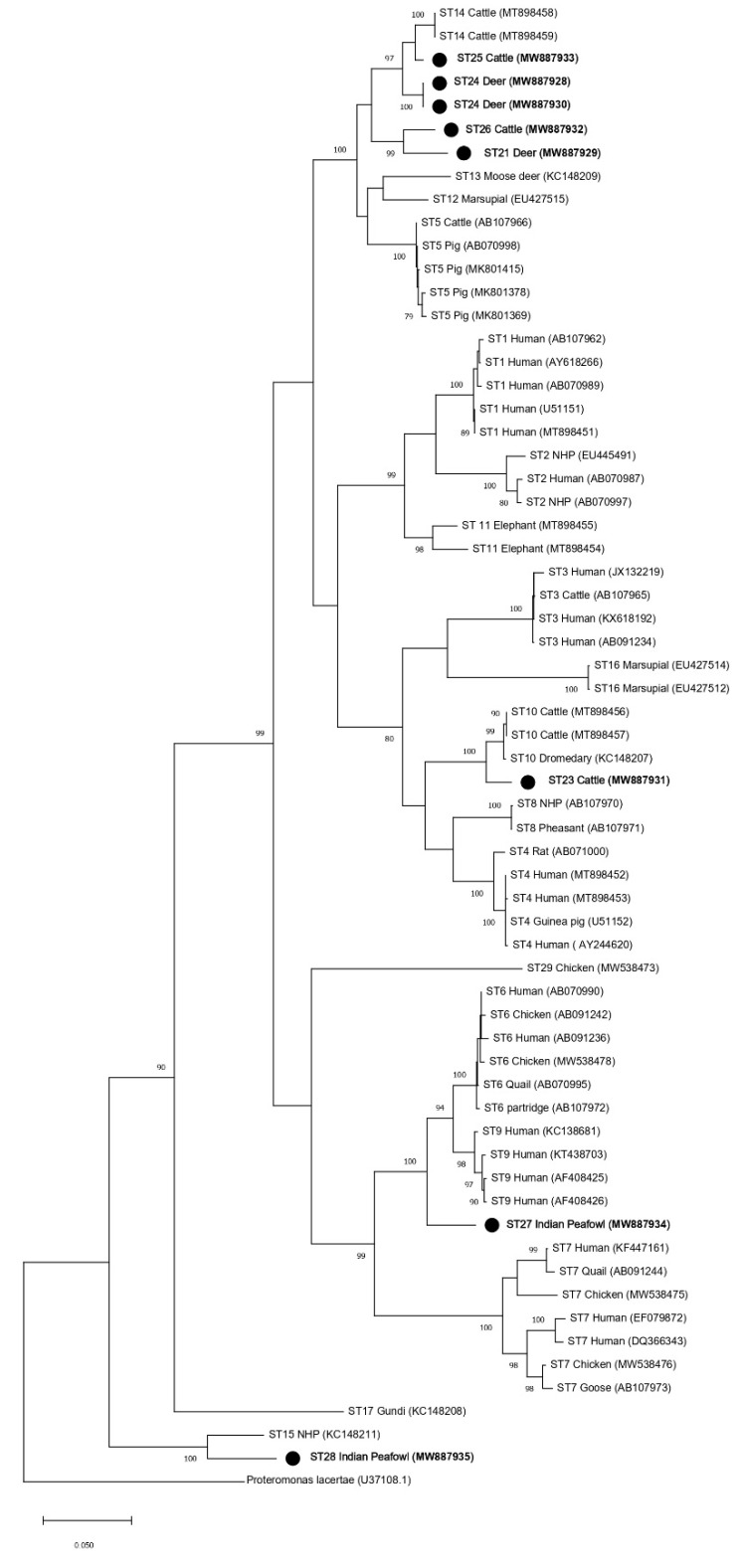
Phylogenetic relationships among *Blastocystis* partial *SSU* rRNA gene sequences of the Santin region generated in the present study (represented with a black filled circle) and representative reference sequences of the accepted subtypes (Table 2). *Proteromonas lacertae* was used as outgroup taxon to root the tree. Analysis was conducted by a maximum likelihood method. Genetic distances were calculated using the Kimura two-parameter model. This analysis involved 64 nucleotide sequences, and there were a total of 568 positions in the final dataset. Bootstrap values lower than 75% are not displayed.

**Figure 7:**
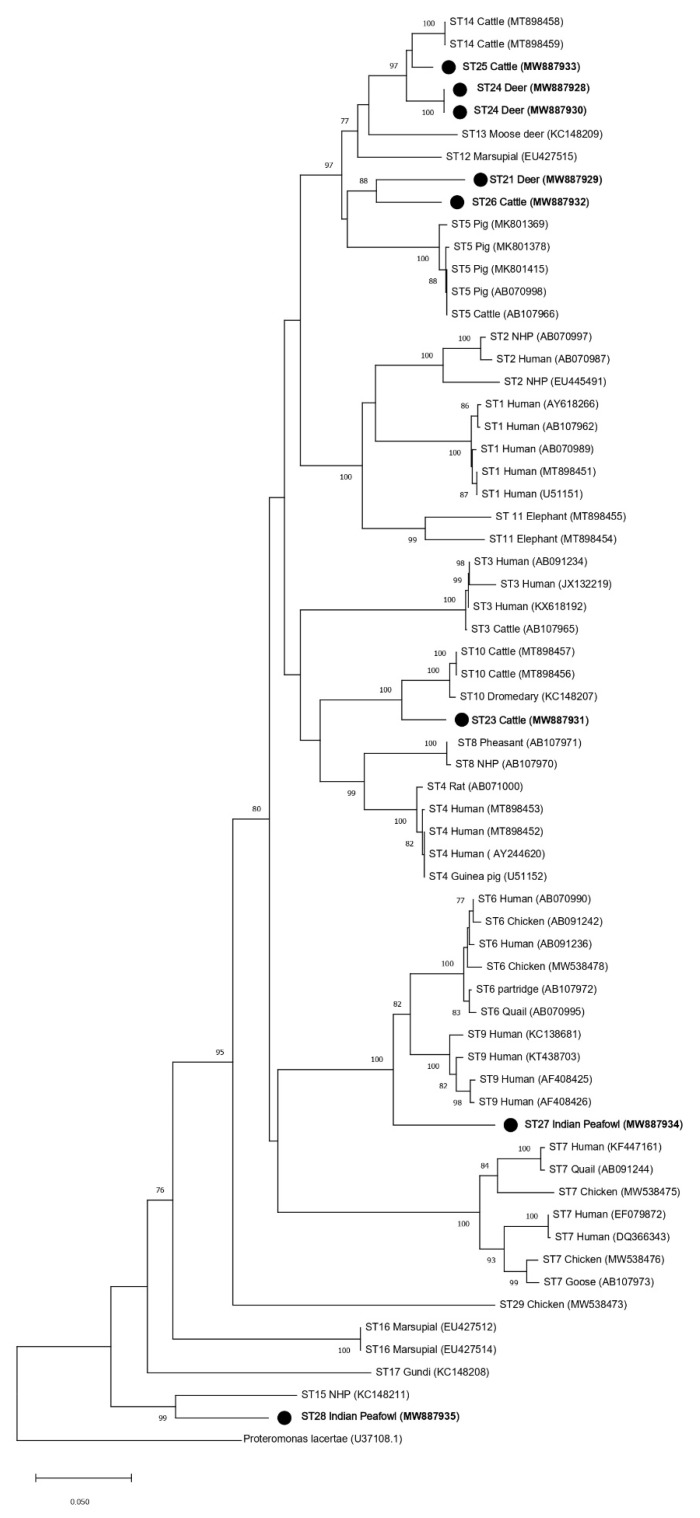
Phylogenetic relationships among *Blastocystis* partial *SSU* rRNA gene sequences of the Santin region sequences generated in the present study (represented with a black filled circle) and representative reference sequences of the accepted subtypes (Table 2). *Proteromonas lacertae* was used as outgroup taxon to root the tree. Analysis was conducted by a neighbor-joining method. Genetic distances were calculated using the Kimura two-parameter model. This analysis involved 64 nucleotide sequences, and there were a total of 568 positions in the final dataset. Bootstrap values lower than 75% are not displayed.

Phylogenetic trees using ML and NJ were also constructed using partial *SSU* rRNA gene sequences of the barcoding region (Figure 4 and Figure 5) and the Santin region, which was used in the initial designation of ST23–ST28 (Figure 6 and Figure 7). The topology of the ML and NJ trees generated using partial gene regions showed similar relationships between ST21, ST23, ST24, ST25, ST26, ST28, and the rest of the subtypes when compared to full-length sequences (Figure 4,Figure 5,Figure 6,Figure 7). Clades formed by ST23, ST27, and ST28 using the barcoding region in ML tree were supported by bootstrap proportions of 99, 92, and 99, respectively (Figure 4). By NJ, bootstrap proportions for ST23, ST27, and ST28 clades were 100, 64, and 100, respectively (Figure 5). Similarly, clades formed by ST23, ST27, and ST28 using the Santin region were all supported by a bootstrap proportion of 100 by ML, respectively (Figure 6) and a bootstrap proportion of 100, 100, and 99 by NJ, respectively (Figure 7). However, ST27 formed a clade with ST9 in the tree for the barcoding region for ML (Figure 4) but basally branched in NJ (Figure 5) and basally branched for both ML and NJ in the Santin region (Figure 6 and Figure 7). Clades formed by ST21 and ST26 were supported by a bootstrap proportion of 80 and 94 using barcoding for ML and NJ, respectively (Figure 4 and Figure 5) and 99 and 88 using the Santin region for ML and NJ, respectively. As observed in full-length analyses, ST25 formed a clade with ST14 using the Santin region with a bootstrap support of 97 for ML and NJ (Figure 6 and Figure 7). For the barcoding region, ST24 and ST25 formed a clade together with bootstrap support of 85 for NJ (Figure 4), but this clade was not supported for ML (Figure 5).

### 3.3. Pairwise Distance

Comparison of pairwise distances for all full-length sequences was also performed to determine average sequence distances between new full-length references and currently accepted subtypes (Table 2). The percentage of average sequence similarity among all subtypes ranged from 78 to 98%. For pairs of subtypes including the new full-length references ST24/ST25, ST14/ST24, ST14/ST25, and ST10/ST23 were all found to share 98% of sequence identity across the full-length *SSU* rRNA gene (Table 3). ST21 and ST26 were found to share 96% of sequence identity across the full-length *SSU* rRNA gene (Table 3). Comparisons of pairwise distances of partial sequences for the barcoding region (Table 4) and Santin region (Table 5) were also performed. The percentage of sequence similarity among all sequences for the barcoding region ranged from 80 to 99%. The barcoding region showed a sequence similarity of ≥96% between several of the new reference sequences and existing subtypes (Table 3). Within the barcoding region, a sequence similarity of 99% was observed between pairs ST24/ST25, ST14/ST24, ST14/ST25, ST10/ST23, and ST21/ST26. The percentage of sequence similarity among all sequences for the Santin region ranged from 66 to 96% (Table 4). Furthermore, pairwise distance comparisons for subtypes at the Santin region exhibited greater degrees of divergence between sequences, with sequence similarities between subtypes observed at 97% only for pair ST25/ST14 and 96% for ST14/ST24 and ST24/ST25. The rest of the pairwise distances were lower than 96% (Table 5).

## 4. Discussion

Current recommendations indicate that new subtype designations should only be given if >80% of the approximately 1800 bp *SSU* rRNA gene has been sequenced and demonstrated to vary from any known subtype by at least 4% across the full-length of the potentially novel sequence [9]. With these proposed guidelines, obtaining full-length reference sequences of the *SSU* rRNA gene of *Blastocystis* is now essential for accurate subtype identification and for determining novel subtypes. Additionally, full-length sequences of the *SSU* rRNA gene allow for the construction of phylogenies that can aide in more accurately describing the relationship between *Blastocystis* subtypes from humans and other animal hosts. However, this requirement has proven difficult to achieve, and several of the up-to 29 proposed subtypes have been named using only partial gene sequences. This issue is highlighted by the fact that subtypes described after 2013, ST18–ST28, were all named using a fragment of the *SSU* rRNA gene [10,20,21]. A recent review of new subtypes ST18–ST26 suggested that official acceptance be withheld for ST21 and ST23–26 until more data including full-length *SSU* rRNA reference sequences can be analyzed, while it was recommended that ST18–ST20 and ST22 be rejected on the basis of appearing to be artifactual sequences [9].

It was recently demonstrated that MinION long read sequencing could be used to successfully produce accurate full-length reference sequences of the *Blastocystis SSU* rRNA gene [15]. This method was validated using both cultured and fecal isolates of *Blastocystis*, and its suitability for the detection of sequences from samples containing both multiple subtypes and multiple variants of the same subtype was demonstrated. MinION sequencing was also successfully employed to produce the first full-length reference sequence for ST11 [16]. In the current study, MinION sequencing was employed to effectively produce full-length *SSU* rRNA reference sequences for the seven *Blastocystis* subtypes for which there are no known published full-length reference sequences (ST21, ST23, ST24, ST25, ST26, ST27, and ST28). The availability of full-length sequences of the *SSU* rRNA gene for all *Blastocystis* subtypes is essential to establish relationships among subtypes.

All subtype sequences generated in this study were produced using samples that were pre-screened using Illumina MiSeq sequencing of the 500 bp region of the *SSU* rRNA gene, which is sometimes referred to as the Santin region [12,13]. The pre-screening of samples via MiSeq was employed to determine both the intra and potential inter-subtype diversity of *Blastocystis* present in samples, as well as to provide comparison sequences for assessment of accuracy. All MinION-generated sequences were found to match their MiSeq counterpart with 99.6–100% similarity observed between sequences generated using the two methods (Table 1). This high degree of similarity between the two sequencing methods is in agreement with previously published reports that compared the two methods and further supports the use of MinION sequencing for reference sequence generation [11,16].

The current recommendation for a new subtype designation states that a phylogenetic analysis should be performed to ensure that new *Blastocystis* subtypes do not nest within any previously known subtypes [9]. This requirement is unclear because all phylogenies are composed of clades nested within larger clades, but we presume to interpret this recommendation to mean strong support for branching demonstrated through high bootstrap support (BS). Thus, phylogenetic analyses were performed using both full-length reference sequences generated in this study via MinION sequencing and reference sequences of established *Blastocystis* subtypes (Table 2). All new subtype sequences reported here did, in fact, form branches that were supported by high bootstrap support (≥96 BS), with the exception of ST25 (45 BS in the ML tree and 93 BS in the NJ tree) (Figure 2 and Figure 3). Low BS for the branching of ST5 from the clade containing ST12 and ST13 (19 BS), as well as ST12 and ST13 (24 BS) in the ML tree, was also observed. Thus, the phylogenetic analysis of full-length *SSU* rRNA sequences strongly supported the assignment of subtype designations for ST21, ST23, ST24, ST25, ST26, ST27, and ST28, when using NJ. There was weaker support for ST25 using ML, but this was also observed for previously accepted subtypes using ML.

The addition of new full-length *SSU* rRNA gene reference sequences to the phylogenetic analysis of the *Blastocystis SSU* rRNA gene had mostly subtle effects on the overall topology of the tree when compared to previous full-length analyses (Figure 2 and Figure 3) [15]. However, a notable shift in the branching pattern for ST16 occurred following the inclusion of full-length references for ST21–ST29. Previous phylogenetic analyses that included ST1–ST17 have concluded that ST16 lacks a specific related mammalian lineage [15,22]. However, in the present analyses, ST16 formed a clade with ST3, thus indicating that it may, in fact, share common ancestry with other subtypes of mammalian lineage (Figure 2 and Figure 3). This finding also supports the importance of full-length reference sequences in informing our understanding of the relationships among *Blastocystis* subtypes.

Interestingly, in the full-length *SSU* rRNA gene trees, branching patterns for the new subtypes that were originally identified in ruminants (ST21, ST23, ST24, ST25, and ST26) indicated common ancestry for ST21 and ST26, ST23 and ST10, and ST14, ST24, and ST25 (Figure 2 and Figure 3). ST21, which was originally reported in a waterbuck in China, forms a clade with ST26 that was originally named in cattle in the United States [20,21]. Both ST21 and ST26 have been most frequently identified in cattle, so their shared ancestry may support their host specificity in ruminants [2]. A similar relationship between ST14, ST24, and ST25 is also documented, with ruminants being the most common hosts of these subtypes [2]. ST10 and ST23 branch together and form a clade with ST4 and ST8 (Figure 2 and Figure 3). Both ST10 and ST23 are most commonly reported in cattle, and it has been suggested that ST10 is cattle-adapted subtype [2,20,23]. The data presented here support a shared ancestry for ST10 and ST23 but indicate a more distant relationship to other subtypes common to ruminants. Though ST21, ST23, ST24, ST25, and ST26 form a clade shared with other subtypes commonly reported in mammals, ST27 and ST28 (which were originally reported in birds) do not (Figure 2 and Figure 3) [10]. ST27 forms a clade that includes ST6, ST7 and ST9, and two of those subtypes—ST6 and ST7—are the most common subtypes reported in birds and have been suggested to be host-adapted to birds [2,24]. The full-length *SSU* rRNA tree presented here supports the common ancestry of these potentially avian-adapted subtypes. ST28 forms a clade with ST15 (Figure 2 and Figure 3). ST15, while commonly reported in mammals, appears to be distantly related to other mammalian subtypes [2]. ST28 joins ST15 in representing the two most basally branching subtypes of *Blastocystis*.

Phylogenies were also generated from two *SSU* rRNA gene regions commonly used to subtype *Blastocystis* when studying subtype distribution and frequency in different hosts [13,14]. While it was observed that the topology of the two regions for trees generated using ML and NJ were similar for the relationships of ST21, ST23, ST24, ST25, ST26, and ST28, there was a differences in branching for ST27, which formed an external branch in the barcoding region NJ tree (Figure 5) but branched with ST9 in the barcoding ML tree (Figure 4) and in both NJ and ML for the Santin region tree (Figure 6 and Figure 7). The differences between the two trees could likely be at least in part explained by the levels of variability contained within the two regions, with the Santin region containing more overall variability than the barcoding region (Table 3, Table 4 and Table 5). We wish to avoid the over-analysis of partial sequence trees because it was previously demonstrated that partial *SSU* rRNA gene sequences may be insufficient for the phylogenetic analysis of *Blastocystis* subtypes [15]. However, similar branching patterns between the full-length *SSU* rRNA gene and the Santin region, which was used in the original designation of ST23–ST28, were observed, thus supporting their designation as new subtypes of *Blastocystis*.

As sequence distance is currently used to define subtypes of *Blastocystis*, a comparison of the pairwise distances of all full-length *SSU* rRNA gene sequences was also performed (Table 3, Table 4 and Table 5). In pairwise distance comparisons, ST21 and ST26 were found to have the most similarity with respect to the rest of the subtypes, with 96% of sequence identity across the full-length *SSU* rRNA gene (Table 3). Thus, ST21 and ST26 meet all current recommendations to be named as new subtypes. ST27 shared the most sequence similarity with ST6 and ST9 (95%), and ST28 shared the most sequence similarity with ST15 (93%) (Table 3). Both ST27 and ST28 were found to well-exceed the 4% divergence recommended to name them as new subtypes. However, ST24 and ST25, ST14 and ST24, ST14 and ST25, and ST10 and ST23 were all found to share 98% of sequence identity across the full-length *SSU* rRNA gene (Table 3).

Though ST23, ST24, and ST25 were not found to meet the recommended minimum divergence of 4% across the full-length SSU rRNA gene, we suggest that these subtypes still be considered as valid. These subtype designations are already in use and have been documented in studies involving different hosts and/or countries [9,10]. Observations of these sequences in multiple studies supports their validity as novel subtypes as opposed to experimental artifacts, and discontinuing their use would only add further confusion to assigning new subtypes in the future. Furthermore, these subtypes are frequently identified in both domestic and wild ruminant species, and differentiating between these sequence variants could provide important information on the role of cross-species transmission. These subtypes are easily differentiated using the Santin region of the *SSU* rRNA gene (Table 5). However, it should be noted that ST23, ST24, and ST25 may not be easily distinguished using more conserved regions of the *SSU* rRNA gene such as the barcoding region (Table 4). This is an issue that extends beyond the identification of ST23, ST24, and ST25. For example, the already accepted subtypes ST5, ST12, and ST13 share 96–97% sequence identity with ST14 within the barcoding region, which may make their accurate identification difficult (Table 5). This may contribute to the increased incidence of sequences in GenBank sharing a high degree of identity but being named as any one of these subtypes, e.g., GenBank accession MN526814, which is recorded as ST14 returns BLAST matches for ST5 (99% identity, MK937752) and ST13 (97% identity, MF186700). For the Santin region, only ST14/ST25 were found to exceed the 96% cut-off, sharing a 97% similarity, while the similarity between other pairs was found to be ≤96% (Table 5), thus indicating this region could be better suited for subtyping *Blastocystis* isolates.

The full-length *SSU* rRNA gene reference sequence for ST27 allowed for a comparison of its barcoding region to other barcoding sequences available on GenBank. A Blast search of the first 600 bp of the full-length sequence returned 13 accessions with 98–99% of sequence identity and 95–97% of sequence coverage to ST27, which are listed here in order of shared identity: MK861944, MK861940, MK861941, MK861943, MK861939, MK861937, MK861936, MK861942, MK930361, MK357782, MT661535, MK861938, and MK861946. As all of these sequences also came from peafowl but from China (unpublished), it is likely that they represent other examples of ST27 and expand the range of ST27 to another continent. The MK861944 sequence is listed as a new subtype and shares 99% of identity with ST27. However, some sequences share a 98% identity with ST27 but are given new or already used subtype designations. For example, MT661535 shares 98% of identity with ST27 but is recorded as ST9, and MK930361 shares 98% of sequence identity with ST27 but is recorded as ST18. Because these sequences have no corresponding publication at this time, it is hard to know how the authors arrived at these subtype designations. However, these findings further highlight the need for quality full-length *SSU* rRNA gene references for describing *Blastocystis* diversity and host distribution.

## 5. Conclusions

Historically, new subtypes of *Blastocystis* have been named using both full-length and partial gene sequences of the *SSU* rRNA gene. However, the current recommendation suggests that a nearly full-length sequence be available before a new subtype designation can be given. While the data presented here highlight that different regions of the gene yield different relationships between subtypes based on sequence identity, it is also important to note that the requirement of a full-length sequence may not be an achievable goal for all researchers working in this field. In fact, attempting to procure the longest possible sequence may be contributing to the generation and publication of chimeric sequences, as some of the primers used to generate longer sequences of the gene may lack the specificity of shorter fragment primers. Mixed subtypes within a sample are common but can be difficult to detect using traditional Sanger sequencing [12]. Sanger sequencing produces a consensus of all present sequences, so either poor quality sequences generated from mixed infections or the propensity of forward and reverse primers to amplify different subtypes that are then stitched together into a single chimeric sequence are not easily identified. By comparison, the high sequence depth and stringent bioinformatic processing of MinION-generated sequences can better handle these issues, but this technology may not be widely available to all researchers. As the world of *Blastocystis* sequencing continues to expand to new hosts and regions of the world, these are outstanding issues that will need to be addressed so that all researchers can easily and accurately subtype their isolates. As additional sequences of both known and new subtypes are compiled, our understanding of the relationships within this diverse species complex will likely change and bring with it an enhanced understanding of host specificity, pathogenic potential, and the complex epidemiology of *Blastocystis*.

## Figures and Tables

**Table 1:** Information of *Blastocystis* specimens used in this study including host, geographic origin, and subtype.

Specimen ID/Isolate	Host	Location	*Blastocystis* Subtype (GenBank Accession Number)	Similarity to MiSeq Sequence (%)
1 (Deer4)	White-tailed deer	Maryland, USA	ST24 (MW887928)	100
2 (Deer 79)	White-tailed deer	Maryland, USA	ST21 (MW887929) ST24 (MW887930)	100 100
3 (2817.14 m)	Cattle	Maryland, USA	ST23 (MW887931) ST26 (MW887932)	100 100
4 (2813.20 m)	Cattle	Maryland, USA	ST25 (MW887933)	100
5 (Bird36)	Indian peafowl	Uberlândia, Brazil	ST27 (MW887934) ST28 (MW887935)	99.6 99.8

**Table 2:** *Blastocystis* full-length *SSU* rRNA gene nucleotide sequences used in the construction of phylogenetic trees including information on host, country of origin, isolate/strain, sequence length, and GenBank accession number associated with each sequence. **Bold denotes sequences obtained in this study.**

Subtype	Host	Country	Isolate/Strain	Sequence Length (bp)	GenBank Accession No.
ST1	Human	USA	Nand ^a^	1770	U51151
	Human	Thailand	989	1781	AY618266
	Human	Japan	PJ99-172	1770	AB107962
	Human	N/A	HJ96A-29	1770	AB070989
	Human	USA	1 ^a^	1766	MT898451
ST2	Human	Japan	HJ96-1	1768	AB070987
	NHP	Japan	JM92-2	1768	AB070997
	NHP	Philippines	M24	1721	EU445491
ST3	Human	N/A	HJ96A-26	1719	AB091234
	Human	Senegal	N/A	1755	JX132219
	Human	Singapore	N/A	1769	KX618192
	Cattle	Japan	CJ99-363	1769	AB107965
ST4	Human	Germany	HG00-12	1778	AY244620
	Human	Spain	H-1/3 ^b^	1773	MT898453
	Human	USA	2 ^c^	1772	MT898452
	Guinea pig	USA	NIH:1295:1 ^d^	1778	U51152
	Rat	Japan	RN94-9	1778	AB071000
ST5	Cattle	Japan	CJ99-284	1784	AB107966
	Pig	Austria	L01064_Blast_ST5_99	1749	MK801415
	Pig	Germany	L00855_Blast_ST5_97	1738	MK801369
	Pig	Germany	L00926_Blast_ST5_99	1738	MK801378
	Pig	Japan	SY94-3	1784	AB070998
ST6	Human	N/A	HJ96AS-1	1691	AB091236
	Human	N/A	HJ96AS-1	1741	AB070990
	Chicken	Brazil	96.7	1738	MW538478
	Chicken	N/A	CK92-4	1692	AB091242
	Japanese quail	Japan	QQ93-3	1741	AB070995
	Partridge	Japan	BJ99-310	1741	AB107972
ST7	Human	China	HC05-10	1797	DQ366343
	Human	China	HC06-08	1795	EF079872
	Human	Singapore	B	1819	KF447161
	Chicken	Brazil	96.9	1813	MW538475
	Chicken	Brazil	96.10	1786	MW538476
	Goose	Japan	BJ99-569	1796	AB107973
	Quail	N/A	QQ98-4	1756	AB091244
ST8	NHP	Japan	MJ99-132	1779	AB107970
	Pheasant	Japan	BJ99-319	1779	AB107971
ST9	Human	Japan	HJ00-4	1736	AF408425
	Human	Japan	HJ05-4	1741	KT438703
	Human	Denmark	N/A	1668	KC138681
	Human	Japan	HJ00-5	1737	AF408426
ST10	Cattle	USA	5	1770	MT898456
	Cattle	USA	6a	1770	MT898457
	Dromedary	Libya	CA6	1728	KC148207
ST11	Elephant	USA	4a	1762	MT898454
	Elephant	USA	4b	1763	MT898455
ST12	Marsupial	N/A	MWJ04-41	1772	EU427515
ST13	Mouse deer	UK	Mousedeer	1765	KC148209
ST14	Cattle	USA	6b	1771	MT898458
	Cattle	USA	7	1771	MT898459
ST15	NHP	UK	MA7	1898	KC148211
ST16	Marsupial	N/A	MKJ04-10	1748	EU427512
ST16	Marsupial	N/A	MKJ04-30	1748	EU427514
ST17	Gundi	Libya	Gundi	1983	KC148208
**ST21**	**White-tailed deer**	**USA**	**Deer79**	**1776**	**MW887929**
**ST23**	**Cattle**	**USA**	**2817.14 m**	**1777**	**MW887931**
**ST24**	**White-tailed deer**	**USA**	**Deer4**	**1769**	**MW887928**
**ST24**	**White-tailed deer**	**USA**	**Deer79**	**1770**	**MW887930**
**ST25**	**Cattle**	**USA**	**2813.20 m**	**1782**	**MW887933**
**ST26**	**Cattle**	**USA**	**2817.14 m**	**1785**	**MW887932**
**ST27**	**Indian peafowl**	**Brazil**	**Bird36**	**1729**	**MW887934**
**ST28**	**Indian peafowl**	**Brazil**	**Bird36**	**1808**	**MW887935**
ST29	Chicken	Brazil	96.6	1776	MW538473

NHP: non-human primate; N/A: not available. ^a^ ATCC# 50177; ^b^ Isolate H-1 reported by [13] and reported as specimen ID#3 by [15]; ^c^ ATCC# 50608; ^d^ ATCC# 50578.

**Table 3:** Pairwise distances between *Blastocystis* subtypes full-length *SSU* rRNA gene sequences showing the average number of base substitutions per site. Analyses were conducted using the Kimura 2-parameter model and included 64 nucleotide sequences (Table 2). There were a total of 1953 positions in the final dataset. Red background indicates pairwise distance lower than 0.04.

		Subtypes																								
		1	2	3	4	5	6	7	8	9	10	11	12	13	14	15	16	17	21	23	24	25	26	27	28	29
**Subtypes**	**1**																									
	**2**	0.07																								
	**3**	0.14	0.13																							
	**4**	0.12	0.13	0.09																						
	**5**	0.11	0.11	0.13	0.12																					
	**6**	0.13	0.14	0.15	0.14	0.13																				
	**7**	0.17	0.17	0.18	0.17	0.15	0.12																			
	**8**	0.11	0.12	0.10	0.06	0.12	0.14	0.17																		
	**9**	0.13	0.13	0.15	0.14	0.13	**0.03**	0.11	0.14																	
	**10**	0.13	0.13	0.11	0.08	0.11	0.13	0.17	0.08	0.13																
	**11**	0.06	0.08	0.14	0.12	0.11	0.13	0.16	0.12	0.13	0.12															
	**12**	0.10	0.11	0.13	0.13	0.06	0.12	0.15	0.12	0.12	0.12	0.11														
	**13**	0.11	0.11	0.14	0.13	0.07	0.13	0.16	0.12	0.12	0.12	0.11	0.06													
	**14**	0.10	0.11	0.13	0.12	0.06	0.13	0.16	0.12	0.12	0.11	0.10	0.05	0.05												
	**15**	0.20	0.20	0.18	0.18	0.17	0.17	0.20	0.18	0.16	0.17	0.19	0.17	0.18	0.17											
	**16**	0.15	0.15	0.12	0.12	0.14	0.15	0.18	0.13	0.15	0.13	0.15	0.14	0.15	0.15	0.18										
	**17**	0.17	0.18	0.17	0.18	0.16	0.16	0.20	0.18	0.16	0.18	0.17	0.16	0.17	0.17	0.19	0.18									
	**21**	0.11	0.11	0.13	0.13	0.07	0.12	0.15	0.12	0.12	0.12	0.11	0.06	0.07	0.06	0.18	0.14	0.17								
	**23**	0.13	0.13	0.11	0.08	0.11	0.13	0.17	0.08	0.13	**0.02**	0.12	0.11	0.12	0.11	0.18	0.14	0.17	0.12							
	**24**	0.11	0.11	0.13	0.12	0.06	0.13	0.15	0.12	0.12	0.11	0.10	0.05	0.05	**0.02**	0.16	0.14	0.17	0.06	0.11						
	**25**	0.10	0.10	0.13	0.12	0.06	0.12	0.15	0.12	0.12	0.11	0.10	0.05	0.05	**0.02**	0.17	0.14	0.16	0.06	0.11	**0.02**					
	**26**	0.11	0.10	0.13	0.13	0.06	0.13	0.15	0.12	0.12	0.12	0.11	0.06	0.06	0.05	0.17	0.14	0.16	**0.04**	0.11	0.05	0.05				
	**27**	0.13	0.14	0.16	0.15	0.13	0.05	0.12	0.15	0.05	0.14	0.13	0.13	0.13	0.12	0.18	0.16	0.18	0.12	0.15	0.13	0.12	0.13			
	**28**	0.20	0.20	0.17	0.18	0.16	0.18	0.20	0.19	0.18	0.18	0.18	0.17	0.19	0.17	0.07	0.18	0.18	0.17	0.18	0.17	0.17	0.17	0.19		
	**29**	0.16	0.16	0.17	0.16	0.17	0.17	0.18	0.16	0.16	0.17	0.16	0.17	0.16	0.15	0.22	0.18	0.20	0.16	0.17	0.15	0.15	0.16	0.16	0.20	

**Table 4:** Pairwise distances between *Blastocystis* subtypes barcoding region sequences showing the average number of base substitutions per site. Analyses were conducted using the Kimura 2-parameter model and included 64 nucleotide sequences (Table 2). There were a total of 587 positions in the final dataset. Red background indicates pairwise distance lower than 0.04.

		Subtypes																								
		1	2	3	4	5	6	7	8	9	10	11	12	13	14	15	16	17	21	23	24	25	26	27	28	29
**Subtypes**	**1**																									
	**2**	0.05																								
	**3**	0.09	0.08																							
	**4**	0.09	0.10	0.06																						
	**5**	0.09	0.09	0.10	0.08																					
	**6**	0.14	0.14	0.14	0.14	0.14																				
	**7**	0.18	0.17	0.15	0.17	0.18	0.09																			
	**8**	0.10	0.11	0.05	0.06	0.10	0.13	0.16																		
	**9**	0.14	0.14	0.13	0.11	0.14	**0.03**	0.09	0.12																	
	**10**	0.10	0.11	0.08	0.06	0.11	0.13	0.16	0.05	0.12																
	**11**	0.05	0.07	0.10	0.09	0.09	0.14	0.17	0.11	0.14	0.09															
	**12**	0.10	0.10	0.09	0.09	**0.04**	0.14	0.17	0.09	0.15	0.09	0.09														
	**13**	0.10	0.10	0.10	0.09	0.05	0.13	0.18	0.10	0.14	0.09	0.09	**0.04**													
	**14**	0.08	0.09	0.10	0.07	**0.04**	0.14	0.18	0.10	0.14	0.09	0.08	**0.03**	**0.03**												
	**15**	0.19	0.18	0.15	0.16	0.17	0.15	0.15	0.16	0.16	0.15	0.17	0.15	0.17	0.16											
	**16**	0.12	0.13	0.09	0.09	0.12	0.14	0.18	0.10	0.13	0.10	0.13	0.11	0.11	0.11	0.14										
	**17**	0.18	0.17	0.13	0.14	0.17	0.15	0.16	0.15	0.16	0.15	0.17	0.16	0.16	0.16	0.15	0.14									
	**21**	0.09	0.10	0.10	0.08	0.05	0.12	0.16	0.09	0.14	0.08	0.09	**0.03**	**0.04**	**0.03**	0.16	0.11	0.15								
	**23**	0.11	0.12	0.07	0.06	0.11	0.12	0.18	0.06	0.12	**0.01**	0.10	0.10	0.09	0.09	0.15	0.10	0.15	0.08							
	**24**	0.09	0.10	0.10	0.08	0.04	0.14	0.17	0.10	0.14	0.08	0.08	**0.03**	**0.04**	**0.01**	0.16	0.10	0.16	**0.03**	0.09						
	**25**	0.08	0.09	0.09	0.07	**0.04**	0.13	0.16	0.09	0.13	0.08	0.07	**0.03**	**0.04**	**0.01**	0.16	0.10	0.16	**0.03**	0.09	**0.01**					
	**26**	0.09	0.10	0.10	0.08	0.05	0.14	0.16	0.09	0.14	0.08	0.08	**0.03**	**0.04**	**0.03**	0.17	0.10	0.16	**0.01**	0.08	**0.02**	**0.02**				
	**27**	0.15	0.16	0.14	0.13	0.15	0.05	0.09	0.14	0.05	0.14	0.15	0.16	0.14	0.15	0.17	0.15	0.16	0.15	0.13	0.16	0.15	0.15			
	**28**	0.19	0.17	0.15	0.16	0.16	0.16	0.18	0.16	0.18	0.16	0.17	0.15	0.17	0.16	0.06	0.15	0.15	0.15	0.16	0.16	0.15	0.16	0.19		
	**29**	0.16	0.16	0.15	0.17	0.18	0.16	0.17	0.16	0.16	0.15	0.17	0.16	0.16	0.16	0.20	0.15	0.20	0.16	0.15	0.16	0.16	0.16	0.16	0.18	

**Table 5:** Pairwise distances between *Blastocystis* subtypes Santin region sequences showing the number of base substitutions per site between sequences. Analyses were conducted using the Kimura 2-parameter model and included 64 nucleotide sequences (Table 2). There were a total of 568 positions in the final dataset. Red background indicates pairwise distance lower than 0.04.

		Subtypes																								
		1	2	3	4	5	6	7	8	9	10	11	12	13	14	15	16	17	21	23	24	25	26	27	28	29
**Subtypes**	**1**																									
	**2**	0.11																								
	**3**	0.18	0.22																							
	**4**	0.16	0.18	0.15																						
	**5**	0.17	0.16	0.19	0.17																					
	**6**	0.21	0.21	0.22	0.20	0.20																				
	**7**	0.25	0.27	0.24	0.24	0.22	0.24																			
	**8**	0.18	0.19	0.15	0.08	0.19	0.20	0.25																		
	**9**	0.20	0.21	0.22	0.20	0.19	0.07	0.23	0.20																	
	**10**	0.19	0.18	0.17	0.13	0.17	0.19	0.25	0.13	0.19																
	**11**	0.12	0.14	0.21	0.17	0.18	0.21	0.25	0.19	0.22	0.19															
	**12**	0.18	0.17	0.18	0.15	0.11	0.20	0.24	0.15	0.19	0.14	0.18														
	**13**	0.18	0.17	0.18	0.18	0.12	0.23	0.22	0.17	0.21	0.16	0.18	0.10													
	**14**	0.15	0.16	0.19	0.16	0.10	0.21	0.24	0.17	0.20	0.16	0.15	0.09	0.08												
	**15**	0.31	0.33	0.27	0.29	0.24	0.28	0.32	0.29	0.27	0.29	0.32	0.27	0.28	0.29											
	**16**	0.28	0.28	0.24	0.24	0.24	0.26	0.30	0.26	0.24	0.27	0.27	0.24	0.27	0.26	0.22										
	**17**	0.32	0.32	0.28	0.29	0.24	0.28	0.31	0.29	0.26	0.28	0.30	0.29	0.30	0.28	0.25	0.23									
	**21**	0.16	0.17	0.18	0.17	0.12	0.22	0.21	0.20	0.20	0.20	0.19	0.12	0.13	0.12	0.28	0.23	0.28								
	**23**	0.20	0.17	0.17	0.13	0.15	0.19	0.23	0.13	0.19	0.05	0.18	0.15	0.16	0.17	0.28	0.25	0.29	0.18							
	**24**	0.17	0.18	0.19	0.18	0.09	0.20	0.23	0.18	0.20	0.17	0.16	0.08	0.09	**0.04**	0.28	0.24	0.27	0.11	0.17						
	**25**	0.16	0.17	0.18	0.16	0.09	0.20	0.22	0.16	0.18	0.16	0.15	0.08	0.09	**0.03**	0.28	0.26	0.27	0.11	0.16	**0.04**					
	**26**	0.15	0.18	0.18	0.17	0.09	0.21	0.21	0.18	0.20	0.17	0.17	0.11	0.11	0.10	0.26	0.24	0.28	0.08	0.15	0.09	0.09				
	**27**	0.21	0.24	0.23	0.20	0.22	0.09	0.25	0.21	0.10	0.21	0.23	0.22	0.22	0.21	0.29	0.27	0.30	0.23	0.23	0.22	0.20	0.23			
	**28**	0.29	0.31	0.24	0.27	0.25	0.26	0.29	0.27	0.26	0.29	0.29	0.25	0.28	0.28	0.11	0.23	0.26	0.25	0.28	0.26	0.26	0.25	0.27		
	**29**	0.26	0.26	0.25	0.22	0.26	0.26	0.30	0.23	0.26	0.25	0.26	0.26	0.26	0.25	0.29	0.25	0.34	0.25	0.25	0.25	0.26	0.26	0.26	0.27	

## Data Availability

All relevant data are within the article and its additional files. The sequences data were submitted to the GenBank database under the accession numbers MW887928-MW887935.
